# Effect of Parathyroidectomy Timing on the Successful Resolution of Tertiary Hyperparathyroidism in Kidney Transplant Recipients: A Systematic Review and Meta-Analysis

**DOI:** 10.3390/jcm14175939

**Published:** 2025-08-22

**Authors:** Ioannis Karniadakis, Leandros Stefanopoulos, Charalampos Balomenakis, Georgios Geropoulos, Kyriakos Psarras, Georgios Koimtzis

**Affiliations:** 1 Department of General Surgery, St. George’s University Hospital, London SW9 0QT, UK; ioanniskarniadakis@gmail.com; 2 Department of Electrical and Computer Engineering, Northwestern University, 633 Clark St., Evanston, IL 60208, USA; leandros@northwestern.edu; 3 General Surgery Department, Ulster Hospital, South Eastern Health and Social Care Trust, Dundonald, Belfast BT16 1RH, UK; ch.balomenakis@gmail.com; 4 3rd Department of Surgery, AHEPA University Hospital, School of Medicine, Aristotle University of Thessaloniki, 54642 Thessaloniki, Greece; gerogios.geropoulos@nhs.net (G.G.); psarrask@auth.gr (K.P.); 5 Department of General Surgery, Grange University Hospital, Caerleon Road, Cwmbran NP44 8YN, UK

**Keywords:** parathyroidectomy, kidney transplantation, hyperparathyroidism

## Abstract

**Introduction:** Tertiary hyperparathyroidism following kidney transplantation is a well-recognized complication in patients with pre-existing mineral imbalances due to chronic renal failure. Parathyroidectomy remains the only definitively curative option for tertiary hyperparathyroidism. The optimal timing of parathyroidectomy, before or after transplantation, is debated in the literature. This study aims to assess whether parathyroidectomy timing affects the successful resolution of tertiary hyperparathyroidism in patients with a functional kidney transplant. **Methods:** We conducted a systematic review and meta-analysis of the available literature collating the effect of pre- versus post-transplantation parathyroidectomy on the resolution of tertiary hyperparathyroidism. We compared the follow-up parathyroid hormone and calcium levels of patients subjected to either of these two approaches. **Results:** Three studies were identified, encompassing a total of 223 patients. The meta-analysis of available data yielded no statistically significant difference between pre- and post-kidney transplantation parathyroidectomy in terms of serum parathyroid hormone (SMD −0.19, 95% CI −0.92 to 0.55, *p* = 0.62) and calcium levels (SMD −0.75, 95% CI −2.30 to 0.80, *p* = 0.35). **Conclusions:** We demonstrated no significant difference between pre- and post-transplantation parathyroidectomy when it comes to the treatment of tertiary hyperparathyroidism. This meta-analysis is limited by the small number of studies included, reducing its statistical power. Therefore, additional studies are required to identify the optimal timing of intervention for the effective management of tertiary hyperparathyroidism in kidney transplant recipients.

## 1. Introduction

Hyperplasia of the parathyroid glands and the subsequent development of secondary hyperparathyroidism (HPT) are recognized complications in patients with end-stage kidney disease [1]. In chronic renal failure, the decrease in calcitriol production and changes in vitamin D metabolism, as well as disturbances in calcium (Ca) and phosphorus homeostasis collectively lead to the accelerated synthesis of parathyroid hormone (PTH) [2]. Despite successful kidney transplantation (KT) secondary HPT may persist, thereby, progressing to tertiary HPT [3]. Due to the development of sustained hypercalcemia, tertiary HPT has been identified to increase the rates of post-KT mortality and adversely affect renal graft survival [3,4].

In the modern era of KT, calcimimetic agents are utilized to reduce PTH levels, mitigating the occurrence of post-transplantation hypercalcemia and improving hypophosphatemia [5]. Cinacalcet, approved in the US in 2004 to treat secondary HPT, has been used as an off-label treatment for tertiary HPT in KT recipients [6,7]. However, parathyroidectomy (PTx) remains the only definitively curative option for the treatment of tertiary HPT and has been demonstrated to be superior to cinacalcet therapy in terms of controlling the post-transplantation rates of hypercalcemia [8].

The American Association of Endocrine Surgeons has provided guidance outlining the indications of PTx in patients with secondary and tertiary HPT, which include the following: (1) HPT-associated severe bone pain; (2) spontaneous bone fracture; (3) refractory hypercalcemia and/or hypophosphatemia unresponsive to medical management [9]. Despite being the most successful curative option, PTx timing should carefully be considered by the clinicians, as such a procedure may result in acute surgical complications and persistent hypocalcemia which may affect the patient’s fitness for transplantation [10,11].

The appropriate timing of PTx is a subject of discussion in the literature, as PTx in patients with tertiary HPT may be performed either pre- or post-transplantation. Studies have previously suggested that pre-KT PTx is associated with superior outcomes in terms of graft survival [12,13]. However, the comparative effectiveness of PTx timing on the ultimate resolution of HPT in renal transplant recipients with tertiary HPT remains unclear. The aim of this systematic review and meta-analysis is to assess whether the timing of PTx has an impact on the successful resolution of tertiary HPT in patients with a functional KT.

## 2. Materials and Methods

### 2.1. Literature Search

This systematic review and meta-analysis were conducted according to a protocol that was submitted to the PROSPERO register (registration number: CRD42024533042). The study was undertaken in accordance with the Preferred Reporting Systems for Systematic Reviews and Meta-Analysis (PRISMA) guidelines and the Cochrane Collaboration Handbook for Systematic Reviews of Interventions [14,15].

A thorough search of the literature was performed to identify research articles that investigated the clinical outcomes of pre- versus post-KT PTx in kidney transplant recipients with hyperparathyroidism who failed to respond to medical management. The MEDLINE, Scopus, EMBASE and Cochrane databases were searched for relevant publications until July 2025, according to the PRISMA checklist. Additional research was undertaken to include gray literature studies on the websites of international transplantation, as well as endocrine and renal medicine associations. The search string used the following terms: (“renal” OR “kidney”) AND (“transplant” OR “transplantation”) AND (“parathyroidectomy” OR “hyperparathyroidism”). All key terms were standardized across search engines alongside the use of the appropriate Boolean operators.

Two independent reviewers (I.K. and L.S.) were involved in conducting the first stage of the literature review, evaluating the search results for relevance. Following the initial identification of relevant studies, an exclusion of duplicates was performed. During the second stage of the literature search, all articles were screened by reviewing their respective titles and abstracts. In the last stage of the screening process, a detailed full-text analysis was performed to identify the studies that were ultimately included. Additionally, references from the included full-text articles were also screened for other potential studies for inclusion. In cases of disagreement a third independent reviewer (G.G.) was involved in the decision-making process and the majority’s opinion was involved to aid with the final decision for inclusion. Data extraction was conducted on a pre-drafted data extraction sheet recording study details as well as the desired outcomes.

### 2.2. Inclusion and Exclusion Criteria

Our study included articles that specifically evaluated the effect of PTx timing, which may be performed either before or after KT, on the successful resolution of HPT in KT recipients. The studies that were ultimately included in this meta-analysis had to meet the following criteria: (1) report a direct comparison of the outcomes of pre-versus post-transplantation parathyroidectomy; (2) include only adult patients; (3) all kidney transplant recipients had secondary/tertiary hyperparathyroidism prior to PTx; (4) all patients were subjected to PTx; (5) all patients with a functional renal transplant. Manuscripts that were not yet published, manuscripts with only abstracts available online, and conference abstracts or posters were excluded from this review. No language restriction was applied.

### 2.3. Risk of Bias Assessment of Included Studies

The risk of bias assessment was performed separately by two authors (I.K. and L.S.) with a third author (K.P.) consulted for the resolution of any disagreements.

The individual risk of bias for each study separately was calculated using the Risk of Bias in Non-randomized Studies (ROBINS-I) assessment tool provided by the Cochrane Collaboration [16]. This tool examines seven domains [bias due to the following: (1) confounding; (2) participant selection; (3) classification of interventions; (4) deviations from intended interventions; (5) missing data; (6) measurement of outcomes; (7) selection of reported results and ultimately provides an overall assessment of low, moderate, serious or critical risk of bias for each study included in this meta-analysis. The traffic light and summary of bias plots were created using the Risk of Bias VISualization (ROBVIS) tool, using the the robvisR package (McGuinness & Higgins, 2021) [17].

### 2.4. Data Extraction, Outcomes of Interest, and Statistical Analysis

Data extracted from each study included the title of the publication, the name of the first author, the year of publication, the total number of participants, and the number of participants in each arm of every study, as well as the post-procedural serum Ca and PTH levels.

The statistical analyses were performed using Reviewer Manager 5.4.1 software [Review Manager (RevMan) (computer program) version 5.4.1 Copenhagen: The Nordic Cochrane Center, Denmark, the Cochrane Collaboration, 2020]. The value of *p* < 0.05 was used as the cut-off level for statistical significance. The data in this study is presented as Mean ± Standard Deviation (SD). In the event of high heterogeneity, random effects models were used, and outcomes were presented as standard mean differences (SMDs). The SMD magnitude was interpreted by using Cohen’s recommendations of small (0.2), medium (0.5), and large (0.8) effect sizes. The DerSimonian and Laird estimator was used for the calculation of the heterogeneity variance τ^2^. Heterogeneity was tested with the Cochrane χ^2^ test and the degree of between-study heterogeneity was quantified by the I^2^ statistics and its 95% CI. Heterogeneity was classified as Low (0–40%), Moderate (40–75%), and High (>75%). Serum PTH values are presented in pg/mL, and serum Ca values are presented in mg/dL.

Missing means and SDs were calculated from extracted data reported as median (interquartile range—IQR) or median (minimum to maximum) in accordance with the guidance provided by the Cochrane Collaboration for the handling of missing statistics in the meta-analysis of continuous outcomes [18,19]. As per the guidance, missing Means ± SDs were calculated using the formulas provided by Wan et al. [20].

Potential publication bias was examined using Begg’s funnel plot and calculating the respective Egger’s test. The funnel plots were created using Stata version 17 (StataCorp LLC, College Station, TX, USA).

## 3. Results

### 3.1. Literature Search Yield

The original literature search yielded a total of 1157 records across databases. After excluding duplicate articles, the total number of articles was reduced to 573. After title and abstract screening, the total number of studies dropped further to 17. Of these, 1 article did not have an available text online, and a full-text analysis was performed on 16 articles. 13 studies were further excluded, as either the design, protocol and methods investigated different PICO questions to the one in our study, or comprising conference posters or abstracts.

**Figure 1:**
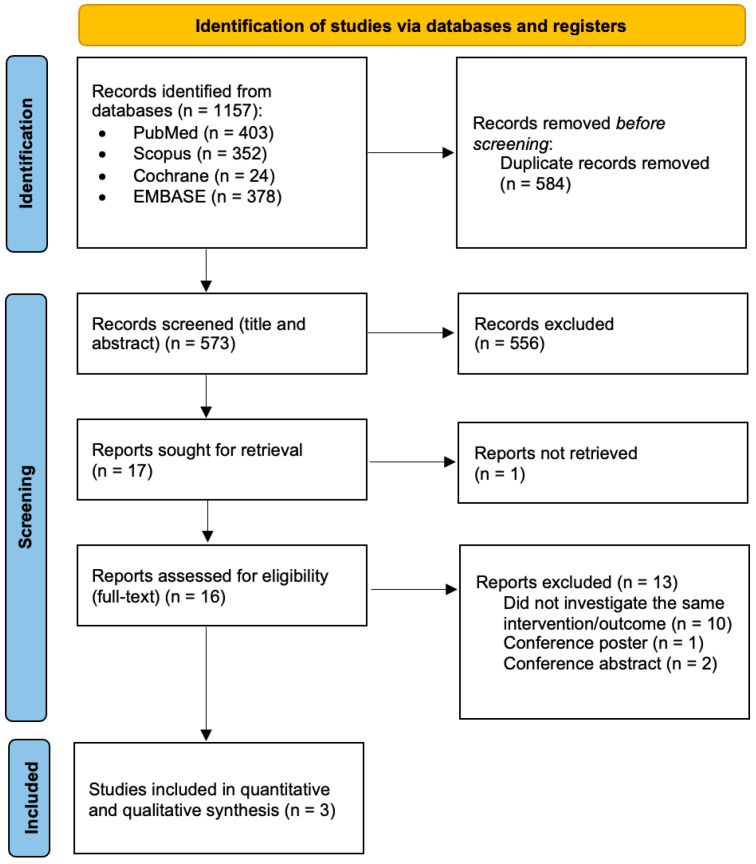
PRISMA flowchart of the study selection process.

A total of three retrospective studies which incorporated 223 total patients (90 in the pre-KT PTx group versus 143 in the post-KT PTx group) were identified as eligible for inclusion in the meta-analysis [21,22,23]. The flowchart of the selection process is shown in Figure 1. Of these research studies, one originated from Japan, one from Turkey, and one from the USA.

### 3.2. Findings of the Systematic Review

All studies ultimately included in this meta-analysis consisted of retrospective reviews [21,22,23]. Okada et al. demonstrated a non-significant difference between serum PTH in the pre- versus post-KT PTx groups [21]. Oruc et al. demonstrated a statistically significant difference in higher serum calcium values in the post-KT group and lower serum PTH values in the pre-KT cohort [22]. Finally, Wang et al. demonstrated no statistically significant differences in terms of PTH and serum Ca between groups [23]. An overview of extracted patient data can be found in Table 1 and Table 2.

### 3.3. Risk of Bias Assessment

**Figure 2:**
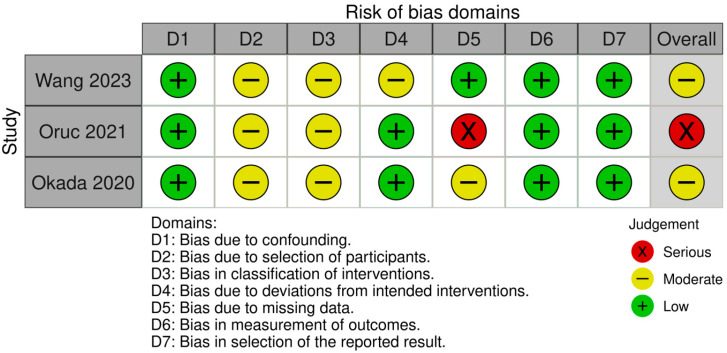
Traffic light plot of individual study risk of bias assessment.

**Figure 3:**
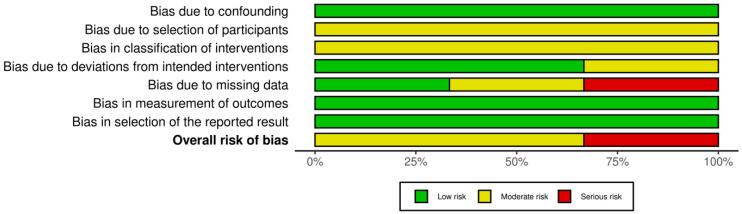
Summary plot of individual risk of bias assessment. Okada et al., 2020 [21], Oruc et al., 2021 [22], Wang et al., 2023 [23].

The summary of risk of bias assessment with regard to individual studies is summarized in Figure 2 and Figure 3.

Two of the studies [21,23] included in the meta-analysis had a moderate overall risk of bias, and a serious overall risk of bias was assessed in the remaining study [22]. All studies demonstrated a low risk of bias due to confounding, and there also were no issues in terms of bias in the measurement of outcomes or selection of the reported result.

Due to the retrospective nature of the studies, the risk of bias was moderate across studies concerning participant selection and classification of intervention. Moderate risk of bias due to classification of interventions was identified in the study by Wang et al., as no subgroup analysis was performed based on whether participants were subjected to parathyroid autotransplantation or subtotal PTx [23]. A serious risk of bias due to missing was identified in the study by Oruc et al. as approximately 21% of participants were excluded due to missing data [22].

### 3.4. Meta-Analysis Outcomes

An overview of the extracted data, as well as the subsequent calculation of the missing means ± SDs is depicted in Table 2. In terms of PTH outcomes, of the studies included, two reported the desired outcomes as median (interquartile range) and one as median (minimum to maximum). Regarding serum Ca outcomes, one study did not report analyzable data, one reported data as median (minimum to maximum) and one as median ± SD.

**Figure 4:**
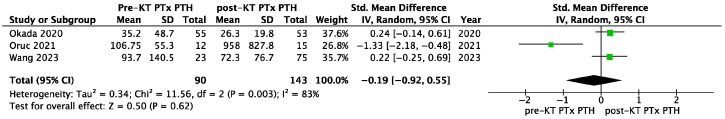
Forest plot for serum PTH on follow-up of patients submitted to PTx pre-KT versus post-KT PTx. KT; kidney transplantation, PTx; parathyroidectomy, PTH, parathyroid hormone. Okada et al., 2020 [21], Oruc et al., 2021 [22], Wang et al., 2023 [23].

Pooled analysis did not demonstrate any statistically significant difference in the timing of PTx on serum PTH between the compared groups (SMD −0.19, 95% CI −0.92 to 0.55, *p* = 0.62). For this calculation, a random-effects model was applied due to the high interstudy statistical heterogeneity (I^2^ = 83%, *p* = 0.003). The outcome of this calculation is demonstrated in Figure 4.

**Figure 5:**
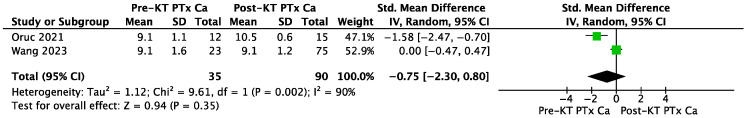
Forest plot of serum Ca on follow-up in the pre-KT versus post-KT PTx groups. KT; kidney transplantation, PTx; parathyroidectomy, Ca; calcium. Oruc et al., 2021 [22], Wang et al., 2023 [23].

Regarding the value of serum Ca on follow-up, the meta-analysis from the available data did not demonstrate a statistically significant difference between the two PTx groups (SMD −0.75, 95% CI −2.30 to 0.80, *p* = 0.35) (Figure 5). This calculation was also performed using a random-effects model as the data again demonstrated high interstudy heterogeneity (I^2^ = 90%, *p* = 0.002).

### 3.5. Publication Bias Assessment

The Funnel plots for the included studies are shown in the Supplementary Material. For our first analysis the Egger’s test yielded *p* value of 0.0011, while for the second one a *p* value of 0.284 indicating the presence of publication bias only in the former one.

## 4. Discussion

To contribute to clinical decision-making, this meta-analysis aimed to compare the effect of pre-KT versus post-KT PTx when it comes to the successful resolution of tertiary HPT. In this study, a total of three publications were identified in the literature which investigated the same research question. All three studies reported serum PTH levels on postoperative follow-up of both patient cohorts in question [21,22,23]. However, only two studies reported post-PTx serum Ca levels [22,23]. In our meta-analysis, no statistically significant difference was demonstrated in the serum PTH levels of patients who underwent PTx before versus after renal transplantation. We also indicated no statistically significant difference in terms of post-PTx serum Ca between the two patient cohorts.

Secondary HPT is a well-known complication of end-stage kidney disease, characterized by phosphorus retention, low serum calcium levels, abnormalities in 1.25-hydroxyvitamin D production and increased serum PTH [24]. KT constitutes the best available treatment for patients with renal failure and has been demonstrated to mitigate the adverse effects of secondary HPT [25]. However, the pre-existing mineral imbalances induced by renal failure may persevere in 30–60% of patients at one year following graft implantation, a condition which has been described as tertiary HPT [26].

Tertiary HPT is hazardous for both patients and kidney allografts. Pihlstrøm et al. conducted a multivariate analysis comparing KT patients with serum PTH > 65 pg/mL to those with normal or low levels and concluded that higher PTH concentrations were associated with a 46% increased risk for all-cause mortality and an 85% increased risk for graft loss, respectively [4]. With regard to the deterioration of graft function, in a longitudinal study of 911 renal transplant patients performed by Araujo et al., it was shown that the presence of tertiary HPT increases the odds of death-censored graft failure, as defined by either the deterioration of the estimated Glomerular Filtration Rate (eGFR) or need for patient return to hemodialysis [27].

Tertiary HPT can be effectively managed either medically, with the use of calcimimetic agents, or through PTx surgery [28,29]. Cinacalcet, the only calcimimetic agent available for human use, facilitates the reduction in circulating PTH by amplifying the sensitivity of the calcium-sensing receptors located in the parathyroid gland [30]. The Kidney Disease: Improving Global Outcomes (KDIGO) CKD Work Group consensus guidelines recommend that, in the post-KT period of the first 12 months, patients with low bone mineral density and an eGFR of > 30 mL/min/1.73 m^2^ should be treated with vitamin D, calcitriol/alfacalcidol, with or without the use of antiresorptive agents [31]. Medical treatment should be guided the presence of mineral bone disease, as identified by abnormal levels of calcium, phosphate, PTH, alkaline phosphatases and 25(OH)D [32]. At the present time, there is insufficient data to inform clinical decision-making beyond the 12-month timeframe, and no data is provided about the role of surgical management in KT recipients [31,32]. Surgical management with PTx is generally performed when hypercalcemia persists despite the administration of medical treatment [13]. Several studies have compared the effectiveness of Cinacalcet versus PTx concerning the successful resolution of tertiary HPT, as defined by the normalization of the patients’ mineral profile. Overall, PTx has been suggested to be superior to Cinacalcet in terms of decreasing serum PTH and Ca levels [33,34].

PTx may be performed either before or after KT in patients with tertiary HPT. The appropriate timing of PTx for the effective management of tertiary HPT has been debated in the literature and at present varies among transplant centers. Some studies have raised concerns regarding the possible adverse effects of PTx on renal graft function, while others do not recommend prophylactic PTx on patients with secondary HPT in the pre-transplant setting [13,35,36].

Of note, this meta-analysis has specific limitations. The retrospective nature of the studies included may introduce a certain degree of bias as demonstrated in our risk of bias assessment. Additionally, differences in the length of follow-up and reported outcome durations, and differences in sample size may have all contributed to the significant interstudy heterogeneity. More specifically, the study conducted by Oruc et al. incorporated a smaller sample size; despite demonstrating a large study effect, this may represent an overestimation of treatment effect due to selection bias, missing data, confounding, or chance. Finally, renal function was not uniformly reported across the included studies of this meta-analysis. Of note, Okada et al. reported median eGFR and interquartile ranges, Oruc et al. reported median creatinine values and minimum to maximum ranges, and Wang et al. reported mean creatinine values alongside their respective SDs. Therefore, a meta-analysis comparing the effect of pre- vs. post-KT PTx or renal function was not feasible while ensuring the statistical accuracy of the study. This meta-analysis is limited by the low number of studies included, which reduces its statistical power.

Further research is warranted to elucidate the effect of PTx timing primarily on the resolution of tertiary HPT in renal transplant patients and secondarily on the post-KT renal function, preferably in the form of Randomized Control Trials.

## 5. Conclusions

This systematic review and meta-analysis aimed to assess the effect of PTx timing on the successful resolution of tertiary HPT. The meta-analysis of a limited number of eligible studies demonstrated that the timing of PTx did not significantly affect serum PTH and calcium levels in KT recipients with tertiary HPT. Further research is required to determine the optimal timing of intervention for the effective management of HPT in this group of patients.

## Figures and Tables

**Table 1:** Characteristics of the individual studies included in the meta-analysis.

Author; Year	Country	Pre-KT PTx *n*	Post-KT PTx *n*	Pre-KT PTx Age	Post-KT PTx Age
Okada; 2020 [21]	Japan	55	53	52.0 (41.0, 60.0) ^1^	52.0 (43.0, 58.0) ^1^
Oruc; 2021 [22]	Turkey	12	15	41.5 (25–60) ^2^	40.8 (24–54) ^2^
Wang; 2023 [23]	USA	23	75	41.3 ± 12.1 ^3^	51.1 ± 10.8 ^3^

KT; kidney transplantation, PTx; parathyroidectomy, *n*; patient number, ^1^ median (IQR), ^2^ median (min-max), ^3^ mean ± SD.

**Table 2:** Tables should be placed in the main text near to the first time they are cited.

Author; Year	Pre-KT PTx PTH	Post-KT PTx PTH	Pre-KT Serum	Post-KT PTx Ca
Okada; 2020 [20]	35.2 ± 48.7	26.3 ± 19.8	n/a	n/a
Oruc; 2021 [21]	106.7 ± 55.3	958.0 ± 827.8	9.1 ± 1.1	10.5 ± 0.6
Wang; 2023 [23]	93.7 ± 140.5	72.3 ± 76.7	9.1 ± 1.6	9.1 ± 1.2

KT; kidney transplantation, PTx; parathyroidectomy, PTH; parathyroid hormone (reported as pg/mL), Ca; calcium (reported as mg/dL), all values reported as mean ± SD.

## Data Availability

The data supporting the findings of this study are available within the article.

## Supplementary Materials

The following supporting information can be downloaded at https://www.mdpi.com/article/10.3390/jcm14175939/s1, Figure S1: Funnel plot of the PTH values meta-analysis; Figure S2: Funnel plot of the calcium values meta-analysis; Table S1: Excluded studies [37,38,39,40,41,42,43,44,45,46].

## Abbreviations

The following abbreviations are used in this manuscript:

HPT	hyperparathyroidism
Ca	calcium
PTH	parathyroid hormone
KT	kidney transplantation
PTx	parathyroidectomy
IQR	interquartile range
SD	standard deviation
