# Supplementary material for: Effect of Parathyroidectomy Timing on the Successful Resolution of Tertiary Hyperparathyroidism in Kidney Transplant Recipients: A Systematic Review and Meta-Analysis
Source: J Clin Med. 2025 Aug 22;14(17):5939. doi: 10.3390/jcm14175939 (PMC12429494; doi:10.3390/jcm14175939)
Supplement: Supplementary file 1 [file jcm-14-05939-s001.zip › jcm-3767025-SI.pdf]

Supplementary material

Manuscript title: Effect of parathyroidectomy timing on the successful resolution of tertiary hyperparathyroidism in kidney transplant recipients: A systematic review and meta-analysis

Manuscript ID: jcm-3767025

Figure S1. Funnel plot of the PTH values meta-analysis

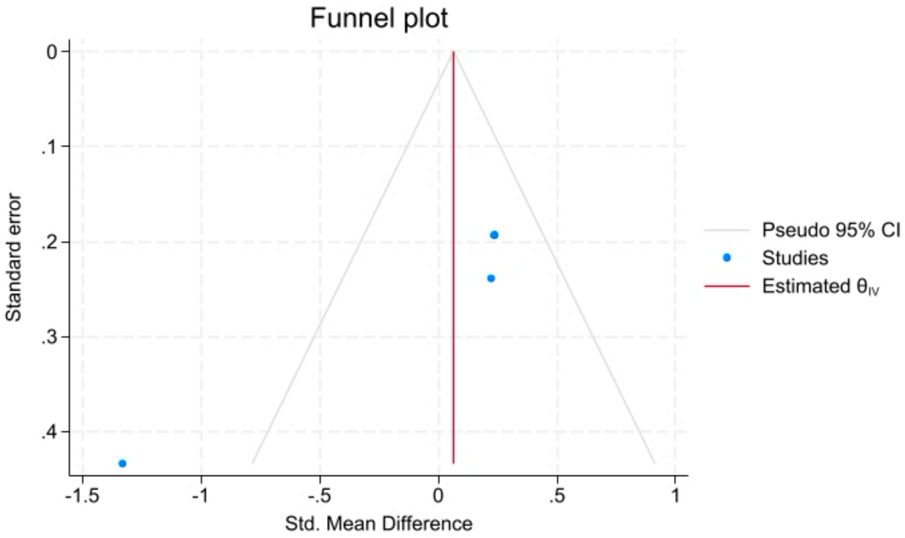

Figure S2. Funnel plot of the calcium values meta-analysis

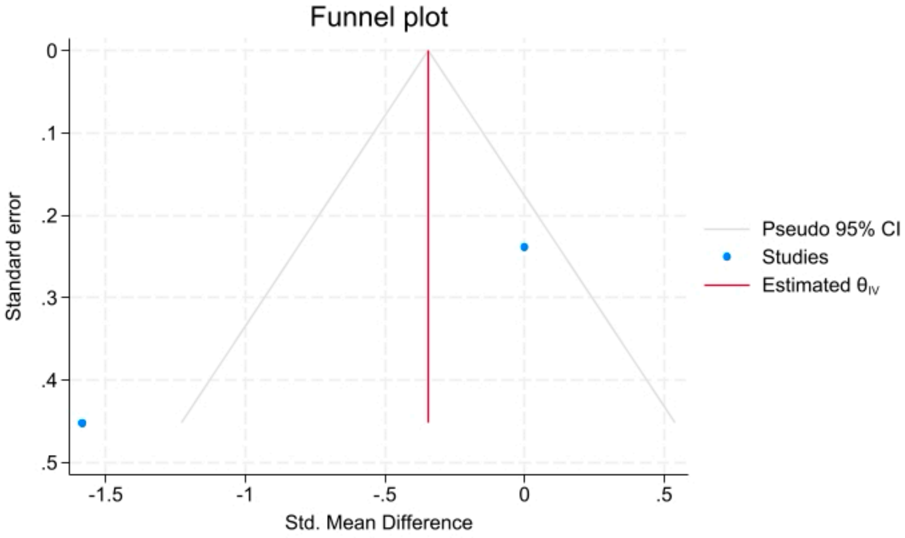

Table S1. Excluded studies

Author; year	Country	Reason for exclusion

Wang; 2024 [1]	USA	Did not investigate the same intervention / outcome
Tsai; 2023 [2]	Taiwan, USA	Did not investigate the same intervention / outcome
Saad; 2020 [3]	USA	Conference abstract
Van de Plas; 2019 [4]	Netherlands	Did not investigate the same intervention / outcome
Kovács; 2019 [5]	Hungary	Did not investigate the same intervention / outcome
Van de Plas; 2018 [6]	Netherlands	Did not investigate the same intervention / outcome
Littbarski; 2018 [7]	Germany	Did not investigate the same intervention / outcome
Littbarski; 2017 [8]	Germany	Conference poster
Gawychowski; 2015 [9]	Poland	Did not investigate the same intervention / outcome
Jeon; 2012 [10]	USA	Conference abstract
Kandil; 2010 [11]	USA	Did not investigate the same intervention / outcome
Evenepoel; 2005 [12]	Belgium	Did not investigate the same intervention / outcome
Wilson; 1971 [13]	USA	Did not investigate the same intervention / outcome

References

1. Wang, R.; Reed, R.D.; Price, G.; Abraham, P.; Lewis, M.; McMullin, J.L.; MacLennan, P.; Killian, C.; Locke, J.E.; Ong, S.; et al. Treatment of Hypercalcemic Hyperparathyroidism After Kidney Transplantation Is Associated With Improved Allograft Survival. *Oncologist* 2024, *29*, e467-e474, doi:10.1093/oncolo/oyad314.
2. Tsai, M.H.; Chen, M.; Liou, H.H.; Lee, T.S.; Huang, Y.C.; Liu, P.Y.; Fang, Y.W. Impact of Pre-Transplant Parathyroidectomy on Graft Survival: A Comparative Study of Renal Transplant Patients (2005-2015). *Med Sci Monit* 2023, *29*, e940959, doi:10.12659/msm.940959.
3. Saad, F.; Wille, K.M.; Ong, S. Parathyroidectomy post kidney transplant: A single center report of short term outcomes. *Journal of Investigative Medicine* 2020, *68*, 671-672, doi:https://dx.doi.org/10.1136/jim-2020-SRM.579.
4. van der Plas, W.Y.; El Moumni, M.; von Forstner, P.J.; Koh, E.Y.; Dulfer, R.R.; van Ginhoven, T.M.; Rotmans, J.I.; Appelman-Dijkstra, N.M.; Schepers, A.; Hoorn, E.J.; et al. Timing of Parathyroidectomy Does Not Influence Renal Function After Kidney Transplantation. *World J Surg* 2019, *43*, 1972-1980, doi:10.1007/s00268-019-04952-w.
5. Kovács, D.; Fedor, R.; Asztalos, L.; Andrási, M.; Szabó, R.P.; Kanyári, Z.; Barna, S.; Nemes, B.; Győry, F. Surgical Treatment of Hyperparathyroidism After Kidney Transplant. *Transplant Proc* 2019, *51*, 1244-1247, doi:10.1016/j.transproceed.2019.03.008.
6. Van Der Plas, W.Y.; Von Forstner, P.; El Moumni, M.; Koh, E.Y.; Dulfer, R.R.; Van Ginhoven, T.M.; Rotmans, J.I.; Appelman-Dijkstra, N.M.; Schepers, A.; Hoorn, E.J.; et al. Impact of parathyroidectomy timing on graft function after kidney transplantation. *Langenbeck's Arch. Surg.* 2018, *403*, 408-409, doi:https://dx.doi.org/10.1007/s00423-018-1664-3.
7. Littbarski, S.A.; Kaltenborn, A.; Gwiasda, J.; Beneke, J.; Arelin, V.; Schwager, Y.; Stupak, J.V.; Marcheel, I.L.; Emmanouilidis, N.; Jäger, M.D.; et al. Timing of parathyroidectomy in kidney transplant candidates with secondary hyperparathryroidism: effect of pretransplant versus early or late post-transplant parathyroidectomy. *Surgery* 2018, *163*, 373-380, doi:10.1016/j.surg.2017.10.016.
8. Littbarski, S.A.; Kaltenborn, A.; Gwiasda, J.; Beneke, J.; Arelin, V.; Schwager, Y.; Stupak, J.V.; Marcheel, I.L.; Emmanouilidis, N.; Jager, M.D.; et al. Pre-transplant versus early or late post-transplant parathyroidectomy in shpt kidney transplant candidates. *Transplant Int.* 2017, *30*, 29-30, doi:https://dx.doi.org/10.1111/tri.13065.
9. Gawrychowski, J.; Mucha, R.; Paliga, M.; Koziołek, H.; Buła, G. Assessment of operative treatment of patients with tertiary hyperparathyroidism after kidney transplantation. *Endokrynol Pol* 2015, *66*, 422-427, doi:10.5603/ep.2015.0052.
10. Jeon, H.J.; Kim, Y.J.; Kim, M.G.; Kwon, H.Y.; Park, J.H.; Yun, I.J.; Ahn, C.; Yang, J. Impact of parathyroidectomy on allograft outcomes in kidney transplantation. *Am. J. Transplant.* 2012, *12*, 376-377, doi:https://dx.doi.org/10.1111/j.1600-6143.2012.04112.x.
11. Kandil, E.; Florman, S.; Alabbas, H.; Abdullah, O.; McGee, J.; Noureldine, S.; Slakey, D.; Zhang, R. Exploring the effect of parathyroidectomy for tertiary hyperparathyroidism after kidney transplantation. *Am J Med Sci* 2010, *339*, 420-424, doi:10.1097/MAJ.0b013e3181d8b6ff.

12. Evenepoel, P.; Claes, K.; Kuypers, D.; Maes, B.; Vanrenterghem, Y. Impact of parathyroidectomy on renal graft function, blood pressure and serum lipids in kidney transplant recipients: a single centre study. *Nephrol Dial Transplant* 2005, *20*, 1714-1720, doi:10.1093/ndt/gfh892.
13. Wilson, R.E.; Hampers, C.L.; Bernstein, D.S.; Johnson, J.W.; Merrill, J.P. Subtotal parathyroidectomy in chronic renal failure: a seven-year experience in a dialysis and transplant program. *Ann Surg* 1971, *174*, 640-654, doi:10.1097/00000658-197110000-00009.
